# Comparison of the National Nutritional Survey in Japan Estimated Individual-Based Nutritional Data and NIPPON DATA80 Food Frequency Questionnaires

**DOI:** 10.2188/jea.JE20090228

**Published:** 2010-05-05

**Authors:** Yasuyuki Nakamura, Nagako Okuda, Tanvir Chowdhury Turin, Akira Fujiyoshi, Tomonori Okamura, Takehito Hayakawa, Yasuhiro Matsumura, Katsuyuki Miura, Hirotsugu Ueshima

**Affiliations:** 1 Cardiovascular Epidemiology, Kyoto Women’s University, Kyoto, Japan; 2 Department of Health Science, Shiga University of Medical Science, Ohtsu, Japan; 3 Department of Preventive Cardiology, National Cardiovascular center, Osaka, Japan; 4 Department of Hygiene and Preventive Medicine, Fukushima Medical University, Fukushima, Japan; 5 Faculty of Health Care, Kiryu University, Kiryu, Japan

**Keywords:** food frequency questionnaires, weighed food records, egg, fish, meat

## Abstract

**Background:**

The National Nutritional Survey in Japan (NNSJ) was initiated in 1946. Using the majority of the participants for NNSJ, the National Survey on Circulatory Disorders has been conducted every 10 year since 1960. We performed a comparative study of the NNSJ80 estimated individual-based nutritional data by comparing those with NIPPON DATA80 food frequency questionnaires (FFQ) data.

**Methods:**

A total of 10 546 community residents from 300 randomly selected districts participated in the both surveys in 1980. At baseline, history, physical, and blood biochemical measurement and a nutritional survey by FFQ were performed individually. From household-based NNSJ80 data, we estimated nutrient intakes of each household member by dividing household intake data proportionally using average intakes by sex and age groups calculated for NNSJ95. We re-categorized NNSJ80 estimated data to correspond to NIPPON DATA80 FFQ categories. Data were analyzed in men and women separately.

**Results:**

Cross tables showed fairly good agreement of the two categories. The majorities of participants situated on the diagonally aligned cells or the next to them. Weighted kappa ranged from 0.152 to 0.241. Spearman’s rank correlation coefficients between the two categories ranged from 0.224 to 0.338, and those between NNSJ80 continuous data and NIPPON DATA80 categorical data ranged from 0.237 to 0.354. All these values have *P* < 0.001.

**Conclusions:**

These results may indicate that the present nutritional estimation method is applicable to, further studies.

The National Nutritional Survey in Japan (NNSJ) was initiated in 1946 under the direction of the supreme commander of the General Headquarters with the main purposes of obtaining factual information on the nutritional health and actual food consumption and food requirements in Japan for emergency food supplies from other countries.^1^ Household-based food consumption data had been collected in order to fulfill the above initial purpose. Recently, the survey has been conducted once every year. When the dietary survey method for the NNSJ was changes to obtain individual-based food intake data, the comparability of data that had been collected by the household-based food weighing method since 1946 was regarded as an issue of high priority.^1^ We have accomplished estimating nutrient intakes of each household member by dividing household intake data of NNSJ80 conducted in 1980 proportionally using average intakes by sex and age groups calculated for NNSJ95.^2^ The average intakes in NNSJ95 were calculated by a combination method of household-based food weighing record and an approximation of proportions by which family members shared each dish or food in the household.

Using the majority of the participants for NNSJ, the National Survey on Circulatory Disorders has been conducted every 10 year in order to obtain cross-sectional data on cardiovascular disease prevalence and risk factors since 1960.^3^ At the survey, history, physical, and blood biochemical measurement and a nutritional survey using food frequency questionnaires (FFQ) were performed in individual participants. A cohort study based on the National Survey on Circulatory Disorders in 1980^4^ has been names as the National Integrated Project for Prospective Observation of Non-communicable Disease and Its Trends in the Aged (NIPPON DATA80).^5^

In the present study, we performed a comparative study of the NNSJ80 estimated individual-based nutritional data by comparing those with NIPPON DATA80 food frequency questionnaires (FFQ) data.

## METHODS

### Participants

The participants in this cohort were those in the 1980 National Survey on Circulatory Disorders.^4^ A total of 10 546 community-based participants aged 30 years and over in 300 randomly selected health districts throughout Japan participated in the survey, which consisted of history-taking, physical examinations, blood tests and self-administered questionnaires on lifestyle, including an essential nutritional survey by the FFQ method. The overall population aged 30 years and over in the 300 participating health districts was 13 771. Therefore, the participation rate of the survey was 76.6% before exclusion. Participants with missing data on FFQ were excluded.

### NIPPON DATA80 baseline examination

At baseline, non-fasting blood samples were obtained. Body mass index (BMI) was calculated as weight (kg) divided by the square of height (m). Baseline blood pressures were measured by trained observers using a standard mercury sphygmomanometer on the right arm of seated participants.

A lifestyle survey was also carried out using self-administered questionnaires which asked about the typical daily consumption of 31 food items.^6^ Egg consumption was coded as ≥2 eggs/day, about 1 egg/day, about 1 egg/2 days, about 1 to 2 eggs/week, and less than once/week. Fish and meat intakes were coded separately as ≥2 times/day, about 1 time/day, about 1 time/2 days, about 1 to 2 times/week, and less than once/week. Participants were asked about their alcohol drinking habit (never, past, occasional, and daily drinkers). Reported information was confirmed by public health nurses through interviews with the study participants regarding food consumption, smoking, drinking habit, and present and past medical histories.

### National Nutritional Survey

Food intake survey by weighed food records in three representative consecutive days were conducted by specially trained dietary interviewers. Dietary interviewers visited participants’ houses at least one during the survey. Weekends and holydays were avoided.

We estimated nutrient intakes of each household member by dividing household intake data of NNSJ80 conducted in 1980 proportionally using average intakes by sex and age groups calculated for NNSJ95.^2^ The average intakes in NNSJ95 were calculated by a combination method of household-based food weighing record and an approximation of proportions by which family members shared each dish or food in the household. Detailed methods were described.^7^

For each person, means of the estimated individual food amount from the three days records were used in the analyses. Egg, fish and meat intakes were estimated in grams per person.

### Data analyses

NIPPON DATA80 FFQ categorized food intakes into 5 groups, from the most frequent to the least frequent one. From NNSJ80, individual-based food intakes in grams were estimated. We re-categorized NNSJ80 data so that they corresponded to NIPPON DATA80 FFQ categories. For example, among total of 5228 women, there were 72, 1405, 1687, 1756 and 308 women in 5 egg intake categories in ≥2 eggs/day, about 1 egg/day, about 1 egg/2 days, about 1 to 2 eggs/week, and less than once/week, respectively. NNSJ80 egg intake data ranged from 250 to 0 g/day. We re-categorized NNSJ80 estimated data so that they corresponded to NIPPON DATA80 FFQ categories from the highest to the lowest egg intake participants. We obtained weighted kappa to evaluated agreement for the categorical data of the two systems for egg, fish and meat intakes. We also obtained Spearman’s rank correlation coefficient between the two categories, and between NNSJ80 continuous data and NIPPON DATA80 categorical data. Data were analyzed in men and women separately.

## RESULTS

Cross tables for comparing NIPPON DATA80 FFQ data on egg, fish, and meat intakes and re-categorized NNSJ80 data on these intakes to correspond to NIPPON DATA80 FFQ categories are shown in Tables 1–3. Upper datum in each cell is frequency and lower one in *italic* is percentage of total. For NNSJ80 data, categories and ranges in g/day for three food groups are shown. Egg data in Table 1 shows fairly good agreement of the two categories; except for the two extreme categories, ie, the most and the least intake categories where a relatively few percent of participants are found, the largest number of participants in each row situated on the diagonally aligned cells. The majority of participants situated on the diagonally aligned cells or the next to them. Comparisons of the two systems of fish and meat intakes in Tables 2 and 3 are not as good as those of Table 1, however, the agreements are relatively fair.

**Table 1.** NIPPON DATA80 FFQ vs NNSJ80 estimates on egg intake in 5228 women and 4127 men

**Women** **NNSJ_est** **range (g)**	**FFQ (egg)**					**Total**	**Men** **NNSJ_est** **range (g)**	**FFQ (egg)**					**Total**
	
	**1**	**2**	**3**	**4**	**5**			**1**	**2**	**3**	**4**	**5**	
**1** 89.0–250.0	10	38	15	8	1	72	**1** 85.7–199.4	24	88	26	14	3	155
	*0.19*	*0.73*	*0.29*	*0.15*	*0.02*	*1.38*		*0.58*	*2.13*	*0.63*	*0.34*	*0.07*	*3.76*

**2** 44.2–88.7	34	609	468	257	34	1402	**2** 45.4–85.6	82	605	431	228	27	1373
	*0.7*	*11.7*	*9.0*	*4.9*	*0.7*	*26.8*		*2.0*	*14.7*	*10.4*	*5.5*	*0.7*	*33.3*

**3** 28.0–44.1	13	455	598	553	70	1689	**3** 29.2–45.3	31	391	397	378	39	1236
	*0.3*	*8.7*	*11.4*	*10.6*	*1.3*	*32.3*		*0.8*	*9.5*	*9.6*	*9.2*	*0.9*	*30.0*

**4** 9.1–27.9	14	263	533	794	152	1756	**4** 7.5–29.1	17	268	356	520	58	1219
	*0.3*	*5.0*	*10.2*	*15.2*	*2.9*	*33.6*		*0.4*	*6.5*	*8.6*	*12.6*	*1.4*	*29.5*

**5** **0–9.0**	1	40	73	144	51	309	**5** 0–7.4	2	18	32	74	18	144
	*0.0*	*0.8*	*1.4*	*2.8*	*1.0*	*5.9*		*0.1*	*0.4*	*0.8*	*1.8*	*0.4*	*3.5*

**Total**	72	1405	1687	1756	308	5228	**Total**	156	1370	1242	1214	145	4127
	*1.4*	*26.9*	*32.3*	*33.6*	*5.9*	*100.0*		*3.8*	*33.2*	*30.1*	*29.4*	*3.5*	*100.0*

Cross table for comparing NIPPON DATA80 food frequency questionnaires (FFQ) data on egg intake and re-categorized NNSJ80 data on egg intake (categories and ranges in g/day) to correspond to NIPPON DATA80 FFQ categories. FFQ (egg) 1 to 5 correspond to egg consumption ≥2 eggs/day, about 1 egg/day, about 1 egg/2 days, about 1 to 2 eggs/week, and less than once/week, respectively. Upper datum in each cell is frequency and lower one in *italic* is percentage of total.

NNSJ_est = estimated data from the National Nutrition Survey.

**Table 2.** NIPPON DATA80 FFQ vs NNSJ80 estimates on fish intake in 5231 women and 4129 men

**Women** **NNSJ_est** **range (g)**	**FFQ (fish)**					**Total**	**Men** **NNSJ_est** **range (g)**	**FFQ (fish)**					**Total**
	
	**1**	**2**	**3**	**4**	**5**			**1**	**2**	**3**	**4**	**5**	
**1** 189.2–505.0	41	87	49	57	6	240	**1** 221.0–792.8	57	133	71	43	8	312
	*0.78*	*1.66*	*0.94*	*1.09*	*0.11*	*4.6*		*1.38*	*3.22*	*1.72*	*1.04*	*0.19*	*7.6*

**2** 105.8–189.0	110	627	494	344	31	1606	**2** 124.6–220.8	149	596	398	255	29	1427
	*2.1*	*12.0*	*9.4*	*6.6*	*0.6*	*30.7*		*3.6*	*14.4*	*9.6*	*6.2*	*0.7*	*34.6*

**3** 68.9–105.7	67	558	579	485	49	1738	**3** 81.8–124.5	83	422	451	322	30	1308
	*1.3*	*10.7*	*11.1*	*9.3*	*0.9*	*33.2*		*2.01*	*10.22*	*10.92*	*7.8*	*0.73*	*31.7*

**4** 26.8–68.8	21	309	573	523	59	1485	**4** 33.6–81.7	23	256	363	281	39	962
					
*0.4*	*5.9*	*11.0*	*10.0*	*1.1*	*28.4*	*0.56*	*6.2*	*8.79*	*6.81*	*0.94*	*23.3*

**5** 0–26.7	1	28	39	76	18	162	**5** 0–33.3	2	18	23	63	14	120
	*0.02*	*0.54*	*0.75*	*1.45*	*0.34*	*3.1*		*0.05*	*0.44*	*0.56*	*1.53*	*0.34*	*2.9*

**Total**	240	1609	1734	1485	163	5231	**Total**	314	1425	1306	964	120	4129
	*4.6*	*30.8*	*33.2*	*28.4*	*3.1*	*100*		*7.6*	*34.5*	*31.6*	*23.4*	*2.9*	*100*

Cross table for comparing NIPPON DATA80 food frequency questionnaires (FFQ) data on fish intake and re-categorized NNSJ80 data on fish intake (categories and ranges in g/day) to correspond to NIPPON DATA80 FFQ categories. FFQ (fish) 1 to 5 correspond to fish consumption ≥ twice/day, about once/day, about once/2 days, about 1 to 2 times/week, and less than once/week, respectively. Upper datum in each cell is frequency and lower one in *italic* is percentage of total.

NNSJ_est = estimated data from the National Nutrition Survey.

**Table 3.** NIPPON DATA80 FFQ vs NNSJ80 estimates on meat intake in 5231 women and 4127 men

**Women** **NNSJ_est** **range (g)**	**FFQ (meat)**					**Total**	**Men** **NNSJ_est** **range (g)**	**FFQ (meat)**					**Total**
	
	**1**	**2**	**3**	**4**	**5**			**1**	**2**	**3**	**4**	**5**	
**1** 131.8–628.7	9	47	42	22	3	123	**1** 158.8–298.5	12	50	41	26	4	133
	*0.17*	*0.9*	*0.8*	*0.42*	*0.06*	*2.35*		*0.29*	*1.21*	*0.99*	*0.63*	*0.1*	*3.22*

**2** 73.1–131.7	46	374	413	271	54	1158	**2** 89.6–158.4	44	348	403	219	30	1044
	*0.88*	*7.15*	*7.9*	*5.18*	*1.03*	*22.14*		*1.07*	*8.43*	*9.76*	*5.31*	*0.73*	*25.3*

**3** 43.1–73.0	46	451	633	526	122	1778	**3** 51.8–89.5	54	392	568	387	69	1470
	*0.9*	*8.6*	*12.1*	*10.1*	*2.3*	*34.0*		*1.3*	*9.5*	*13.8*	*9.4*	*1.7*	*35.6*

**4** 15.9–43.0	23	244	585	652	185	1689	**4** 15.1–51.7	21	225	405	466	111	1228
	*0.4*	*4.7*	*11.2*	*12.5*	*3.5*	*32.3*		*0.5*	*5.5*	*9.8*	*11.3*	*2.7*	*29.76*

**5** 0–15.8	0	44	103	218	118	483	**5** 0–14.9	2	29	54	128	39	252
	*0*	*0.84*	*1.97*	*4.17*	*2.26*	*9.23*		*0.05*	*0.7*	*1.3*	*3.1*	*0.9*	*6.1*

**Total**	124	1160	1776	1689	482	5231	**Total**	133	1044	1471	1226	253	4127
	*2.4*	*22.2*	*34.0*	*32.3*	*9.2*	*100*		*3.2*	*25.3*	*35.6*	*29.7*	*6.1*	*100*

Cross table for comparing NIPPON DATA80 food frequency questionnaires (FFQ) data on meat intake and re-categorized NNSJ80 data on meat intake (categories and ranges in g/day) to correspond to NIPPON DATA80 FFQ categories. FFQ (meat) 1 to 5 correspond to fish consumption ≥ twice/day, about once/day, about once/2 days, about 1 to 2 times/week, and less than once/week, respectively. Upper datum in each cell is frequency and lower one in *italic* is percentage of total.

NNSJ_est = estimated data from the National Nutrition Survey.

Weighted kappa and Spearman’s rank correlation coefficients are shown in Table 4. Weighted kappa ranged from 0.152 to 0.241. Weighted kappa for egg is the best in men and women among three food items. Spearman’s rank correlation coefficients between the two categories ranged from 0.224 to 0.338, and those between NNSJ80 continuous data and NIPPON DATA80 categorical data ranged from 0.237 to 0.354. All these values have *P* < 0.001.

**Table 4.** Comparison between NIPPON DATA80 FFQ data and NNSJ80 Estimates

	**Egg**		**Fish**		**Meat**	
			
	**Women**	**Men**	**Women**	**Men**	**Women**	**Men**
**Weighted Kappa**	0.241	0.225	0.152	0.163	0.183	0.168
***P***	<0.001	<0.001	<0.001	<0.001	<0.001	<0.001
**Spearman *r* for Category**	0.338	0.322	0.226	0.224	0.281	0.254
***P***	<0.001	<0.001	<0.001	<0.001	<0.001	<0.001
**Spearman r for Gram Intake**	0.354	0.338	0.237	0.251	0.291	0.270
***P***	<0.001	<0.001	<0.001	<0.001	<0.001	<0.001

Weighted kappa to evaluated agreement for the categorical data of the two systems for egg, fish and meat intakes, and Spearman’s rank correlation coefficient between the two categories, and between NNSJ80 continuous data and NIPPON DATA80 categorical data are shown.

## DISCUSSION

Strength of NNSJ are, (1) it is the nation-wide, population based study and its sample is representative; (2) the survey was started more than 60 years ago in 1946; (3) it is performed annually; and (4) it uses three days weighed recording method for dietary survey. Although household-based food consumption data had been collected in order to fulfill the initial purpose, food intake data for individual participants were not available until 1995. When the dietary survey method for the NNSJ was changed to obtain individual-based food intake data, the comparability of data that had been collected since 1946 was needed badly.^1^ The estimation method we used in the present study to obtain nutrient intakes of each household member was done by dividing household intake data of NNSJ90^8^ proportionally using average intakes by sex and age groups calculated for NNSJ95.^2^^,^^7^^,^^9^

FFQ are an attractive method in epidemiologic studies because of their low respondent burden and ease of administration.^10^ The use of FFQ in estimating food groups and nutrient intake is based on the frequency with which a fixed list of foods of predetermined portion sizes is consumed over an extended period of time. This method relies heavily on memory, and the questions posed to respondents are open to interpretation.^10^ Another approach commonly employed to determine nutrient intake is 24-hour food intake recall method (24HR). 24HR consists of obtaining information on food and fluid intake for the previous day or previous 24 hours. 24HR is based on the assumption that the intake described is typical of daily intake. Although 24HR is one of the easiest methods for collecting information regarding the participant’s intake, it is prone to error: (1) participant may not be able to recall the foods eaten; (2) participant may not be able to estimate the amounts of each food eaten; (3) information given may not be sufficiently specific; (4) participant may not be telling the truth; (5) intake during the previous 24 hours may not be representative of the usual individual intake.^11^ The third method is the use of weighed food records. In contrast to FFQ, weighed food records allow more precise determination of portion sizes, do not rely on memory and are not limited to selection from a predetermined list of foods. Weighed food records are not very practical in large epidemiologic studies, however, because they require extensive participant training, have a high respondent burden and require lengthy data entry by trained personnel.^10^

The National Health and Nutrition Examination Survey (NHANES) is a survey research program conducted by the National Center for Health Statistics (NCHS) to asses the health and nutritional status of adults and children in the United States. In NHANES, 24HR was used mainly.^12^ The uniqueness of NNSJ is its use of weighed food records method in a nation-wide large-scale survey.

The evaluation results of the present study showed fairly good agreement between the NIPPON DATA80 FFQ and NNSJ80 estimations. The present data of weighted kappa to evaluated agreement for the categorical data of the two systems for egg, fish and meat intakes showed fairly good agreement. Our Spearman’s rank correlation coefficients between the NNSJ80 continuous data and NIPPON DATA80 FFQ data ranged within those of previously reported results. For instance, Sasaki et al. compared the FFQ data of the Japan Public Health Center-Based Prospective Study Cohort I with dietary records for food group.^13^ The crude Spearman’s rank correlation coefficient for egg in men was 0.28, and 0.43 in women, and ours were 0.338 for men and 0.354 for women.

### Limitations of the study

First, household-based food consumption data were collected in NNSJ80, and we estimated nutrient intakes of each household member by dividing household intake data of NNSJ80 conducted in 1980 proportionally using average intakes by sex and age groups calculated for NNSJ95. Thus, we do not have data to directly compare with FFQ of NIPPON DATA80. Second, we performed a comparative study between quantitative data of NNSJ80 and frequency data of FFQ of NIPPON DATA80, both categorized into 5 groups. Comparison between the two data of different quality is expected to result in not good agreement. Third, we surveyed a limited number of essential nutritional components, egg, fish and meat, by the food-frequency method.

Understanding that any FFQ contains its own inherent problems,^10^ and the presently obtained results were within the ranges of previously reported results, it may be concluded that the nutritional estimation method used in the present study is applicable to further studies.
